# Impact of Metastatic Lymph Nodes on Survival of Patients with pN1-Category Esophageal Squamous Cell Carcinoma: A Long-Term Survival Analysis

**DOI:** 10.1245/s10434-024-15019-z

**Published:** 2024-02-19

**Authors:** Kexun Li, Kunyi Du, Changding Li, Wenwu He, Simiao Lu, Kun Liu, Chenghao Wang, Xin Nie, Yongtao Han, Yunchao Huang, Qifeng Wang, Lin Peng, Xuefeng Leng

**Affiliations:** 1 Department of Thoracic Surgery, Sichuan Clinical Research Center for Cancer, Sichuan Cancer Hospital & Institute, Sichuan Cancer Center, Affiliated Cancer Hospital of University of Electronic Science and Technology of China, Chengdu, China; 2 Department of Thoracic Surgery I, Key Laboratory of Lung Cancer of Yunnan Province, Yunnan Cancer Hospital, The Third Affiliated Hospital of Kunming Medical University, Kunming, China; 3 Department of Radiation Oncology, Sichuan Cancer Hospital & Institute, University of Electronic Science and Technology of China (UESTC), Chengdu, China; 4 Radiation Oncology Key Laboratory of Sichuan Province, Chengdu, China

**Keywords:** Esophageal squamous cell carcinoma, Lymphadenectomy, Esophagectomy, Lymph node, Lymph node metastasis, pN1 category

## Abstract

**Background:**

The morbidity and mortality rates of esophageal squamous cell carcinoma (ESCC) are high in China. The overall survival (OS) of patients with ESCC is related to lymph node (LN) metastasis (LNM). This study aimed to discuss the impact of metastasis in LN stations on the OS of patients with pathologic N1 (pN1) ESCC.

**Methods:**

Data were obtained from the Esophageal Cancer Case Management database of Sichuan Cancer Hospital and Institute (SCCH-ECCM). Additionally, data of patients with pN1-category ESCC collected between January 2010 and December 2017 were retrospectively analyzed.

**Results:**

Data from 807 patients were analyzed. The median OS of the patients with one metastatic LN (group 1) was 49.8 months (95 % confidence interval [CI], 30.8–68.9 months), whereas the OS of those with two metastatic LNs (group 2) was only 33.3 months (*P* = 0.0001). Moreover, group 1 did not show a significantly longer OS than group 2.1 (patients with 2 metastatic LNs in 1 LNM station; *P* = 0.5736), but did show a significantly longer OS than group 2.2 (patients with 2 metastatic LNs in 2 LNM stations; *P* < 0.0001). After propensity score-matching, the 5-year survival rate for group 1 was 28 %, whereas that for group 2 was 14 % (*P* = 0.0027).

**Conclusions:**

The OS for the patients with one metastatic LN in one LNM was not significantly longer than for the patients with two metastatic LNs in one LNM station. Patients with one LNM station had a significantly longer OS than those with two LNM stations. Thus, the number of LNM stations is a significant determinant of OS in pN1 ESCC.

**Supplementary Information:**

The online version contains supplementary material available at 10.1245/s10434-024-15019-z.

Global Cancer statistics have shown that esophageal squamous cell carcinoma (ESCC) has high morbidity and mortality rates, with higher occurrence rates among men than among women.^1^ Older age,^2^ smoking, and alcohol consumption are important risk factors.^3–5^ The treatment methods for ESCC depend on the tumor stage and location, histologic type, performance status, and comorbidities.

Surgery, radiotherapy, and chemotherapy are the standard treatment methods for ESCC.^6–10^ However, a diagnosis confirmed at an advanced stage of ESCC poses treatment challenges for many patients with ESCC.^1^ In addition, due to the delayed diagnosis and poor therapeutic effect of existing treatment methods, the 5-year overall survival (OS) rate is only approximately 30 %.^1,2^

Each of the currently available treatment methods works differently. For example, radiotherapy uses high-energy electron beams to shrink the tumor and destroy it.^11^ In contrast, chemotherapy involves using drugs as infusions or pills to reduce tumor size and inhibit the tumor’s growth.^12^ Immunotherapy activates the immune system to recognize and destroy tumor cells.^13^

With advances in technology and research, gene therapy, tumor vaccines, and other new techniques are being developed to treat esophageal cancer.^14,15^ However, surgery through radical tumor resection continues to be the cornerstone of comprehensive treatment for ESCC.^6,7,9,16^ Lymph node (LN) metastasis (LNM) poses a significant challenge to the curative effect of the standard treatment options.^17–20^

Currently, the Union for International Cancer Control/American Joint Committee on Cancer (UICC/AJCC) tumor-node-metastasis (TNM) staging and the Japan Esophageal Society (JES) system are the internationally used criteria for cancer staging systems.^9,21^ According to the UICC/AJCC TNM staging, pathologic N1 (pN1) is defined as one or two metastatic LNs. However, as per the 11th Japanese Classification of Esophageal Cancer, based on tumor location, different LN stations are divided into groups: N1, N2, and N3.^9,21^ The two systems differ in terms of the LN (N) category. Unlike UICC/AJCC, the JES system focuses more on metastasis of the LN location.^9,21^ The main purpose of this retrospective study was to discuss the impact of metastasis in different LN stations on the OS of patients with pN1-category ESCC.

## Methods

### Study Design and Patients

Data from 2957 patients with ESCC treated at Sichuan Cancer Hospital (SCCH) from January 2010 to December 2017 were retrospectively reviewed. Data and medical records of the patients were obtained from the database of the hospital’s Esophageal Cancer Case Management (SCCH-ECCM database). The data extracted included demographics (sex and age), tumor factors such as pathologic disease stage (T and N categories), TNM stage, location and grade, clinical disease stage (lymphovascular invasion, nerve invasion, LNM, metastasis in LN station), and clinical treatment method (radical resection).

At the discretion of individual surgeons and based on patient characteristics, 797 patients underwent right transthoracic esophagectomies with two- or three-field LN dissection. Overall, 579 patients underwent the Mckeown method, 218 patients underwent the Ivor-Lewis method, and 669 patients underwent intraoperative thoracic duct ligation. The clinical staging of each patient was discussed by experts before treatment and surgery. After surgery, the surgeon separated the specimens to obtain the LNs, which were named according to the guidelines. The final pathologic results were interpreted by two pathologists and re-signed by another pathologist. The disease stage was classified according to the UICC/AJCC eighth-edition TNM staging system.

Regarding adjuvant treatment, 127 (15.7 %) patients received postoperative adjuvant chemoradiotherapy (CRT), 308 (38.0 %) patients received postoperative adjuvant chemotherapy (CT), and 17 (2.1 %) patients received postoperative radiotherapy (RT). Hereafter, we refer to this collectively as surgery plus postoperative CT or RT/CRT. The specific types of drugs and their doses were determined based on individual patient characteristics and discussed by experts before treatment. Details of the clinical treatment methods are shown in Table S1.

**Fig. 1:**
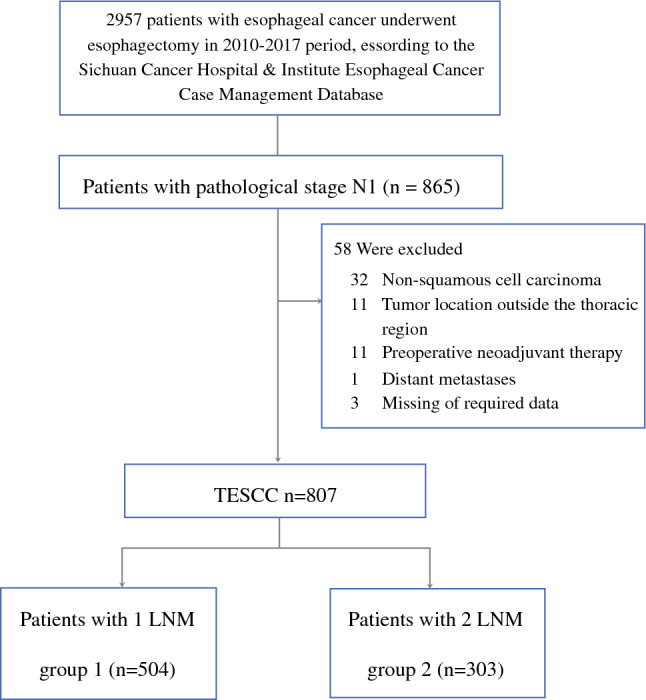
CONSORT diagram showing patient selection. CONSORT, Consolidated Standards of Reporting Trials; TESCC, thoracic esophageal squamous cell carcinoma; LNM, lymph node metastasis

The inclusion criteria for the analysis specified (1) patients who underwent esophagectomy in our hospital and (2) patients classified as pN1. The exclusion criteria ruled out patients who had (1) pathology-confirmed non-squamous cell carcinoma, (2) extra-thoracic tumor location, (3) preoperative neoadjuvant therapy, (4) distant metastasis of tumor, and (5) missing data (Fig. 1).

Patients were followed up once every 3 months for the first 2 years, then once every 6 months thereafter for 3 to 5 years. Overall survival (OS) was defined from the month and year of surgery to death or last follow‑up visit in April 2022.

All procedures performed in this study were in accordance with the Declaration of Helsinki as revised in 2013. Due to the retrospective nature of the study, the Ethics Committee waived consent from patients.

### Criterion of Adverse Events and Characteristic

The patients were divided into two groups: group 1 (patients with 1 metastatic LN according to pathologic results) and group 2 (patients with 2 metastatic LNs). The patients in group 2 were further divided into two subgroups: group 2.1 (patients with metastasis in 1 LN station) and group 2.2 (patients with metastasis in 2 LN stations).

### Statistical Analysis

Categorical variables are presented as percentages. Results were calculated using the chi-square test or Fisher’s exact test. The OS rate was calculated based on the time from the month and year of surgery to the date of death or last follow-up evaluation. Hazard ratios (HRs) and 95 % confidence intervals (CIs) were calculated. Independent OS risk factors were identified using univariate Cox regression analyses. Cox proportional hazards regression models were used to assess the impact of all baseline covariates on outcomes. Kaplan–Meier curves were created using GraphPad Prism 9 (GraphPad Software, San Diego, CA, USA) to evaluate the observation system. Log-rank tests were used to describe the median at specific time points in 95 % CI.

Furthermore, two comparable groups of patients (groups 1 and 2; groups 1 and 2.1; or groups 1 and 2.2) were created using propensity score-matching (PSM) and adjusting unbalanced covariates. For PSM, the caliper was set to a width of 0.02, and the 1:1 closest-match method was used. The PSM procedure analyzed all the variables in the baseline profile (sex, age, pathologic differentiation grade, lymphovascular invasion, nerve invasion, tumor location, pathologic T category, eighth-edition TNM stage, thoracic surgery, abdominal surgery, and clinical treatment method).

A *P* value lower than 0.05 was considered statistically significant. Statistical analyses were performed using the Statistical Package for Social Sciences (SPSS) software version 23.0 (SPSS, Chicago, IL, USA).

## Results

### Clinical Outcomes

For the analysis, 807 patients with pN1-category ESCC were selected. Group 1 included 504 (62.5 %) patients with one metastatic LN, and group 2 included 303 (37.5 %) patients with two metastatic LNs. Group 2.1 included 94 (11.6 %) patients, and group 2.2 included 209 (25.9 %) patients.

In terms of age, 765 (94.8 %) of the patients were younger than 75 years, and 42 (5.2 %) were 75 years of age or older. Furthermore, 662 (82.0 %) of the patients were males, and 145 (18.0 %) were females. Stage III or IV disease was recorded for 754 (93.4 %) of the patients (Table 1). Statistical differences were observed between groups 1 and 2 in terms of lymphovascular invasion (*P* = 0.028) and eight TNM stages (*P* = 0.02). After PSM, the two groups were comparable.

**Table 1:** Demographic characteristics of groups 1 and 2

Characteristic	Before PSM		*P* value	After PSM		*P* value
	Group 1 *n* (%)	Group 2 *n* (%)		Group 1 *n* (%)	Group 2 *n* (%)	
Sex			0.916			0.222
Male	414 (82.1)	248 (81.8)		255 (85.6)	244 (81.9)	
Female	90 (17.9)	55 (18.2)		43 (14.4)	54 (18.1)	
Median age: years (range)			0.687			0.168
<75	479 (95.0)	286 (94.4)		288 (96.6)	281 (94.3)	
≥75	25 (5.0)	17 (5.6)		10 (3.4)	17 (5.7)	
Pathologic differentiation grade			0.157			0.754
Well-differentiated (G1)	91 (18.1)	40 (13.2)		34 (11.4)	40 (13.4)	
Moderately differentiated (G2)	210 (41.7)	127 (41.9)		128 (43.0)	124 (41.6)	
Poorly or not differentiated (G3)	203 (40.3)	136 (44.9)		136 (45.6)	134 (45.0)	
Lymphovascular invasion			0.028			0.344
Yes	76 (15.1)	64 (21.1)		51 (17.1)	60 (20.1)	
No	428 (84.9)	239 (78.9)		247 (82.9)	238 (79.9)	
Nerve invasion			0.754			0.752
Yes	92 (18.3)	58 (19.1)		54 (18.1)	57 (19.1)	
No	412 (81.7)	245 (80.9)		244 (81.9)	241 (80.9)	
Tumor location			0.362			0.840
Upper	138 (27.4)	70 (23.1)		67 (22.5)	70 (23.5)	
Middle	251 (49.8)	164 (54.1)		157 (52.7)	160 (53.7)	
Lower	115 (22.8)	69 (22.8)		74 (24.8)	68 (22.8)	
Pathologic T category			0.841			0.574
T1/T2	138 (27.4)	81 (26.7)		74 (24.8)	80 (22.8)	
T3/T4	366 (72.6)	222 (73.3)		224 (75.2)	218 (73.2)	
8th-Edition TNM stage			0.02			0.838
II	41 (8.1)	12 (4.0)		13 (4.4)	12 (4.0)	
III/IV	463 (91.9)	291 (96.0)		285 (95.6)	286 (96.0)	
Thoracic surgery			0.175			0.934
MIE	241 (47.8)	130 (42.9)		125 (41.9)	126 (42.3)	
OE	263 (52.2)	173 (57.1)		173 (58.1)	172 (57.7)	
Abdominal surgery			0.864			0.734
MIE	191 (37.9)	113 (37.3)		112 (37.6)	108 (36.2)	
OE	313 (62.1)	190 (62.7)		186 (62.4)	190 (63.8)	
Clinical treatment method			0.435			0.458
Surgery alone	217 (43.1)	139 (45.9)		129 (43.3)	138 (46.3)	
Surgery + postoperative CT or RT/CRT	287 (56.9)	164 (54.1)		169 (56.7)	160 (53.7)	

*PSM* propensity score-matching, *TNM* tumor-node-metastasis, *MIE* minimally invasive esophagectomy, *OE* open esophagectomy, *CT* chemotherapy, *RT* radiotherapy, *CRT* chemoradiotherapy

### Survival

**Fig. 2:**
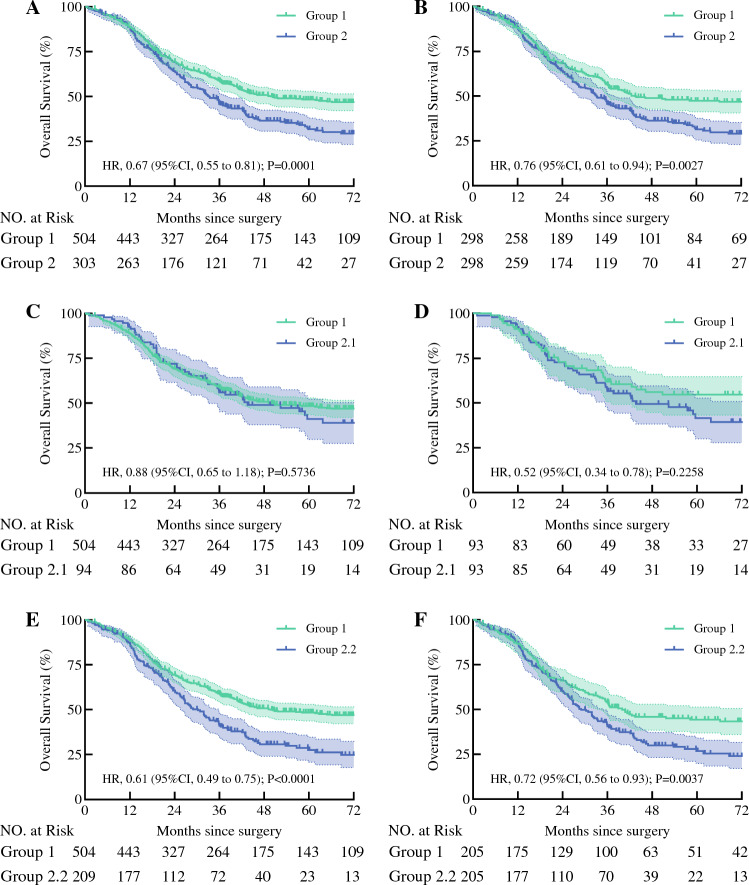
**A** Overall survival curve of patients with metastasis in one lymph node (LN) and patients with metastasis in two LNs before propensity score-matching. **B** Overall survival curve of patients with one LN metastasis and patients with two LN metastases after score-matching. **C** Overall survival curve of patients with one LN metastasis and patients with two LN metastases in one LN station before propensity score-matching. **D** Overall survival curve of patients with one LN metastasis and patients with two LN metastases in one LN station after propensity score-matching. **E** Overall survival curve of patients with one LN metastasis and patients with two LN metastases in two LN stations before propensity score-matching. **F** Overall survival curve of patients with one LN metastasis and patients with two LN metastases in two LN stations after propensity score-matching

**Fig. 3:**
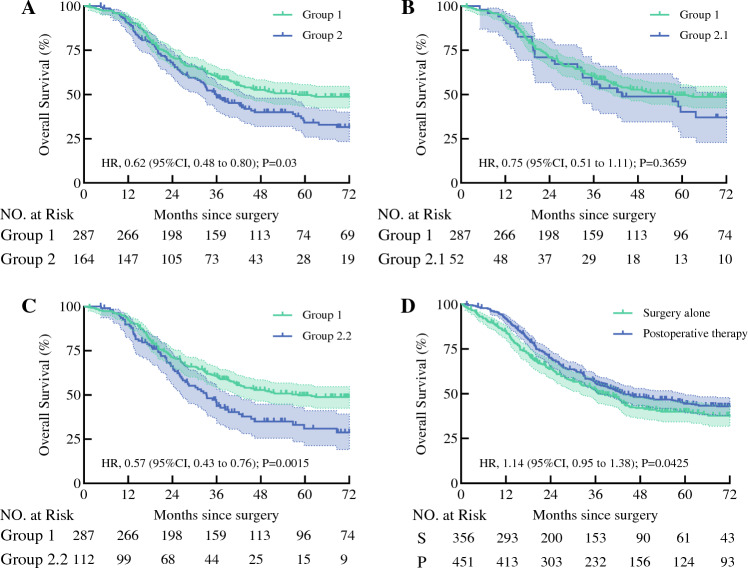
**A** Overall survival curve of analyzed adjuvant therapy patients with one lymph node (LN) metastasis and patients with two LN metastases. **B** Overall survival curve of analyzed adjuvant therapy patients with one LN metastasis and patients with two LN metastases in one LN station. **C** Overall survival curve of analyzed adjuvant therapy patients with one LN metastasis and patients with two LN metastases in two LN stations. **D** Overall survival curve of analyzed patients between surgery alone and postoperative therapy

After a median follow-up time of 65.6 months, the median OS of 807 patients was 41.9 months (95 % CI 36.8–47.0 months). The median OS of the patients in group 1 was 49.8 months (95 % CI 30.8–68.9 months), whereas the OS of the patients in group 2 was 33.3 months (95 % CI 28.3–38.3 months). The 1-, 3-, and 5-year OS rates were 88 %, 52 %, and 28 %, respectively, for the group 1 patients, whereas these values were 87 %, 40 %, and 14 %, respectively, for the group 2 patients (HR, 0.67; 95 % CI 0.55–0.81; *P* = 0.0001; Fig. 2A). Subgroup analyses showed no statistically significant difference in OS between groups 1 and 2.1 (HR, 0.88; 95 % CI 0.65–1.18; *P* = 0.5736; Fig. 2C). However, the OS was significantly longer for the patients in group 1 than for those in group 2.2 (HR, 0.61; 95 % CI 0.49–0.75; *P* < 0.0001; Fig. 2E). Because postoperative adjuvant therapy has a significant impact on OS, relevant subgroup analyses were further performed (Fig. 3). Details of postoperative adjuvant therapy are shown in Tables S1 and S2.

A significant difference in the OS was observed between the patients in groups 1 and 2 after PSM analyses. The 3- and 5-year survival rates for the patients in group 1 were 50 % and 28 %, respectively, whereas those for the patients in group 2 were 40 % and 14 %, respectively (HR, 0.76; 95 % CI 0.61–0.94; *P* = 0.0027; Fig. 2B). In comparison, the 3- and 5-year OS rates for the patients in group 2.1 were 53 % and 20 %, respectively (HR, 0.52; 95 % CI 0.34–0.78; *P* = 0.2258; Fig. 3D). No significant difference in OS was observed between groups 1 and 2.1 after PSM. However, a significant difference in the OS was observed between the patients in groups 1 and 2.2 after PSM analyses (HR, 0.72; 95 % CI 0.56–0.93; *P* = 0.0037; Fig. 3F).

Factors significantly affecting OS after esophagectomy were identified using univariate analyses. These factors included sex (*P* < 0.001), age (*P* = 0.016), tumor grade (*P* = 0.075), lymphovascular invasion (*P* = 0.002), nerve invasion (*P* = 0.03), pathologic T category (*P* < 0.001), TNM stage according to the UICC/AJCC eighth edition (*P* = 0.009), clinical treatment method (*P* = 0.01), number of metastatic LNs (*P* = 0.003), and number of metastatic LN stations (*P* < 0.001; Table 2). Multivariate analyses showed that sex (*P* < 0.001), age (*P* = 0.007), tumor grade (*P* = 0.016), lymphovascular invasion (*P* = 0.026), pathologic T category (*P* < 0.001), and clinical treatment method (*P* = 0.001) were crucial factors affecting 5-year OS after esophagectomy (Table 2).

**Table 2:** Uni- and multivariate Cox regression analyses for factors affecting patient survival

Variables	Univariate			Multivariate		
	HR	95 % CI	*P* value	HR	95 % CI	*P* value
Sex						
Male	Ref.			Ref.		
Female	0.470	(0.328–0.673)	<0.001	0.462	(0.319–0.668)	<0.001
Age (years)						
<75	Ref.			Ref.		
≥75	1.793	(1.114–2.886)	0.016	1.932	(1.193–3.128)	0.007
Pathologic differentiation grade			0.075			0.016
Well-differentiated (G1)	Ref.			Ref.		
Moderately differentiated (G2)	1.442	(0.982–2.118)	0.062	1.539	(1.041–2.277)	0.031
Poorly or not differentiated (G3)	1.553	(1.063–2.269)	0.023	1.764	(1.196–2.602)	0.004
Lymphovascular invasion						
Yes	Ref.			Ref.		
No	0.664	(0.512–0.862)	0.002	0.738	(0.566–0.964)	0.026
Nerve invasion						
Yes	Ref.			Ref.		
No	0.745	(0.571–0.971)	0.03	0.953	(0.724–1.256)	0.735
Tumor location			0.079			
Upper	Ref.					
Middle	1.071	(0.821–1.397)	0.611			
Lower	0.783	(0.567–1.081)	0.137			
Pathologic T category						
T1/T2	Ref.			Ref.		
T3/T4	2.956	(1.393–6.272)	<0.001	1.938	(1.427–2.632)	<0.001
8th-Edition TNM stage						
II	Ref.			Ref.		
III/IV	2.726	(1.289–5.766)	0.009	1.766	(0.798–3.909)	0.161
Thoracic surgery						
MIE	Ref.					
OE	1.076	(0.863–1.342)	0.515			
Abdominal surgery						
MIE	Ref.					
OE	1.086	(0.865–1.364)	0.476			
Clinical treatment method						
Surgery alone	Ref.			Ref.		
Surgery + postoperative CT or RT/CRT	0.752	(0.606–0.934)	0.01	0.696	(0.559–0.866)	0.001
No. of LNMs						
1	Ref.			Ref.		
2	1.391	(1.120–1.727)	0.003	1.266	(0.914–1.753)	0.156
No. of LN stations						
1	Ref.			Ref.		
2	1.584	(1.272–1.973)	<0.001	1.166	(0.835–1.630)	0.367

*HR* hazard ratio, *CI* confidence interval, *TNM* tumor-node-metastasis, *MIE* minimally invasive esophagectomy, *OE* open esophagectomy, *CT* chemotherapy, *RT* radiotherapy, *CRT* chemoradiotherapy, *LN* lymph node, *LNM* LN metastasis

## Discussion

Medical records in the SCCH-ECCM database of the patients who underwent esophagectomy for ESCC were retrospectively analyzed. Based on the grouping of the patients and the pathologic status, our results indicated that the patients with one metastatic LN had significantly longer OS than those with two sites (*P* = 0.0001). However, the OS for the patients with one metastatic LN station was not significantly longer than for the patients with two metastatic LNs in one metastatic LN station (*P* = 0.5736). Moreover, the patients with one metastatic LN had a significantly longer OS than the patients with two metastatic LNs in two LN stations (*P* < 0.0001). These findings remained after PSM, showing no significant difference in the OS between the patients in groups 1 and 2 (*P* = 0.0027) and no significant difference between the patients in groups 1 and 2.1 (*P* = 0.2258). However, a significant difference was observed in the OS between the patients in groups 1 and 2.2 (*P* = 0.0037). In addition, the number of metastatic LNs and the stations of metastatic LNs exhibited significant effects (*P* = 0.003 vs. *P* < 0.001) after multivariate analyses. We believe that metastasis in LN stations is one of the crucial predictors affecting OS after esophagectomy. Thus, we can infer that for patients with pN1 ESCC, the number of metastatic LN stations can lower the OS compared with metastatic LNs.

The current standard treatment method for ESCC includes primarily comprehensive treatment through surgery.^7,9,16,21^ Patients may receive chemotherapy, chemoradiotherapy, or immunotherapy combined with chemoradiotherapy before esophagectomy.^7,16,22,23^ After esophagectomy, additional immunotherapy is considered.^24^ However, the extent of lymphadenectomy in esophagectomy still is a topic of continued discussion.^18–20,25,26^ Considering the pattern of metastasis in ESCC, LN stations have certain clinical value and are gaining increased importance.^8,27^

The TNM staging criteria of the JES system are being progressively developed and enhanced, and the transition from the 11th to the 12th Japanese Classification of Esophageal Cancer highlights the shift from the location-based classification to the number-based classification akin to UICC/AJCC TNM staging.^9^ However, TNM staging using the AJCC/UICC system emphasizes the number of resected metastatic LNs and LN stations.^21^ The data in this study focused not only on the LNM number but also on the LNM stations. It was based on the pN1 status identified using the AJCC/UICC staging system. Although the OS for the patients with one metastatic LN was higher than for those with two metastatic LNs, if two metastatic LNs were located in the different LN stations, this lowered the OS. However, the OS did not show a statistically significant difference in the patients with two metastatic LNs in one metastatic LN station. This further suggests a scope for improvement in the staging system.

Several studies have confirmed that different LN stations have distinct clinical values.^8,27,28^ Ma et al.^28^ showed that the resection of subcarinal and left tracheobronchial LNs has a high clinical value.^28,29^ Furthermore, subcarinal LNM has been shown to decrease the OS of patients. According to the JES system, subcarinal LNM was classified as the pN2 category for upper, middle, and lower thoracic ESCC.^9^ Another example is the left tracheobronchial LN station. Although LN resection of this station can improve OS, because of the high difficulty level associated with the procedure and the potential risk of serious complications, even life-threatening effects resulting from improper management, the necessity of lymphadenectomy in all stations is debatable.^29^ Thus, the factors of LNs that affect OS are complex and multi-sourced. Nevertheless, relevant studies and our research currently confirm that systematic LN dissection is necessary to accurately diagnose the pathological stage of LNs and improve the prognosis of survival in patients.

Our study had limitations. First, although numerous studies on the pattern of LN metastasis have been conducted, the precise pattern of LN metastasis remains uncertain. The importance of each LN station in OS still is controversial and heterogeneous. Second, although the size of the samples from high-volume thoracic surgery centers was relatively large, this was a single-center retrospective analysis. Third, due to heterogeneity, after PSM, the sample size for statistical analysis decreased by hundreds of cases. Future studies using data from multicenter prospective clinical trials are warranted for further assessment of the LN stations, clinical value, and TNM staging.

## Conclusions

The OS for the patients with one LNM was not significantly longer than for the patients with two LNs in one metastatic LN station. Moreover, patients with one metastatic LN station had a significantly longer OS than those with two LN stations. Thus, the number of metastatic LN stations is a significant determinant of OS for patients with pN1-category ESCC.

### Supplementary Information

Below is the link to the electronic supplementary material.

Supplementary file1 (DOCX 16 kb)

Supplementary file2 (DOCX 21 kb)
