# Supplementary material for: Impact of Metastatic Lymph Nodes on Survival of Patients with pN1-Category Esophageal Squamous Cell Carcinoma: A Long-Term Survival Analysis
Source: Ann Surg Oncol. 2024 Feb 19;31(6):3794–802. doi: 10.1245/s10434-024-15019-z (PMC11076366; doi:10.1245/s10434-024-15019-z)
Supplement: Supplementary file 1 — Supplementary file1 (DOCX 16 kb) [file 10434_2024_15019_MOESM1_ESM.docx]

| **Supplementary Table 1**. Details of clinical treatment modalities between the 1 and 2 groups. | | | | | | |
| --- | --- | --- | --- | --- | --- | --- |
| Characteristic | Before PSM | | *P* value | After PSM | | *P* value |
|  | Group 1 | Group 2 |  | Group 1 | Group 2 |  |
| Clinical treatment modality |  |  | 0.717 |  |  | 0.448 |
| Surgery alone | 217  (43.1%) | 139  (45.9%) |  | 129  (43.3%) | 138  (46.3%) |  |
| Surgery plus postoperative RT | 11  (2.1%) | 5  (1.6%) |  | 11  (3.7%) | 5  (1.7%) |  |
| Surgery plus postoperative CT | 192  (38.1%) | 116  (38.3%) |  | 112  (37.6%) | 112  (37.6%) |  |
| Surgery plus postoperative CRT | 84  (16.7%) | 43  (14.2%) |  | 46  (15.4%) | 43  (14.4%) |  |

CT: chemotherapy; CRT: chemoradiotherapy; RT: radiotherapy.
