# Supplementary material for: Impact of Metastatic Lymph Nodes on Survival of Patients with pN1-Category Esophageal Squamous Cell Carcinoma: A Long-Term Survival Analysis
Source: Ann Surg Oncol. 2024 Feb 19;31(6):3794–802. doi: 10.1245/s10434-024-15019-z (PMC11076366; doi:10.1245/s10434-024-15019-z)
Supplement: Supplementary file 2 — Supplementary file2 (DOCX 21 kb) [file 10434_2024_15019_MOESM2_ESM.docx]

| **Supplementary Table 2**. Univariate and multivariate Cox regression analysis for clinical treatment modalities affecting patient survival | | | | | | |
| --- | --- | --- | --- | --- | --- | --- |
| Variables | Univariate | | | Multivariate | | |
|  | HR | 95% CI | *P* | HR | 95% CI | *P* |
| Sex |  |  |  |  |  |  |
| Male | Ref. | | |  | Ref. |  |
| Female | 0.470 | (0.328-0.673) | <0.001 | 0.46 | (0.318-0.665) | <0.001 |
| Age, years |  |  |  |  |  |  |
| <75 | Ref. | | |  | Ref. |  |
| ≥75 | 1.793 | (1.114-2.886) | 0.016 | 1.987 | (1.225-3.224) | 0.005 |
| Pathologic differentiation grade |  |  | 0.075 |  |  |  |
| Well G1 | Ref. | | |  |  |  |
| Moderate G2 | 1.442 | (0.982-2.118) | 0.062 |  |  |  |
| Poor or undifferentiated G3 | 1.553 | (1.063-2.269) | 0.023 |  |  |  |
| Lymphovascular invasion |  |  |  |  |  |  |
| Yes |  | Ref. |  |  | Ref. |  |
| No | 0.664 | (0.512-0.862) | 0.002 | 0.718 | (0.551-0.936) | 0.014 |
| Nerve invasion |  |  |  |  |  |  |
| Yes |  | Ref. |  |  | Ref. |  |
| No | 0.745 | (0.571-0.971) | 0.03 | 0.956 | (0.727-1.258) | 0.749 |
| Tumor location |  |  | 0.079 |  |  |  |
| Upper | Ref. | | |  |  |  |
| Middle | 1.071 | (0.821-1.397) | 0.611 |  |  |  |
| Lower | 0.783 | (0.567-1.081) | 0.137 |  |  |  |
| Pathological T category |  |  |  |  |  |  |
| T1/T2 |  | Ref. |  |  | Ref. |  |
| T3/T4 | 2.956 | (1.393-6.272) | <0.001 | 1.864 | (1.375-2.526) | <0.001 |
| 8th TNM stage |  |  |  |  |  |  |
| Ⅱ |  | Ref. |  |  | Ref. |  |
| Ⅲ/IV | 2.726 | (1.289-5.766) | 0.009 | 1.69 | (0.763-3.74) | 0.196 |
| Thoracic surgery |  |  |  |  |  |  |
| MIE |  | Ref. |  |  |  |  |
| OE | 1.076 | (0.863-1.342) | 0.515 |  |  |  |
| Abdominal surgery |  |  |  |  |  |  |
| MIE |  | Ref. |  |  |  |  |
| OE | 1.086 | (0.865-1.364) | 0.476 |  |  |  |
| Clinical treatment modality |  |  | 0.007 |  |  | 0.001 |
| Surgery alone |  | Ref. |  |  |  |  |
| Surgery plus postoperative RT | 0.252 | (0.093-0.680) | 0.007 | 0.231 | (0.085-0.626) | 0.004 |
| Surgery plus postoperative CT | 0.833 | (0.659-1.052) | 0.125 | 0.801 | (0.633-1.014) | 0.065 |
| Surgery plus postoperative CRT | 0.684 | (0.493-0.949) | 0.023 | 0.599 | (0.43-0.834) | 0.002 |
| The number of LNM |  |  |  |  |  |  |
| 1 |  | Ref. |  |  | Ref. |  |
| 2 | 1.391 | (1.120-1.727) | 0.003 | 1.178 | (0.853-1.627) | 0.32 |
| The number of LN station |  |  |  |  |  |  |
| 1 |  | Ref. |  |  | Ref. |  |
| 2 | 1.584 | (1.272-1.973) | <0.001 | 1.23 | (0.884-1.71) | 0.219 |

CI: confidence interval; CT: chemotherapy; CRT: chemoradiotherapy; RT: radiotherapy; HR: hazard ratio; LN: lymph node; MIE: minimally invasive esophagectomy; OE: open esophagectomy; RT: radiotherapy; TNM: tumor, node, metastasis.
